# Ab initio prediction of semiconductivity in a novel two-dimensional Sb_2_X_3_ (X= S, Se, Te) monolayers with orthorhombic structure

**DOI:** 10.1038/s41598-021-89944-4

**Published:** 2021-05-14

**Authors:** A. Bafekry, B. Mortazavi, M. Faraji, M. Shahrokhi, A. Shafique, H. R. Jappor, C. Nguyen, M. Ghergherehchi, S. A. H. Feghhi

**Affiliations:** 1 Department of Radiation Application, Shahid Beheshti University, Tehran, Iran; 2 Department of Physics, University of Antwerp, Groenenborgerlaan 171, 2020 Antwerp, Belgium; 3 Chair of Computational Science and Simulation Technology, Institute of Photonics, Department of Mathematics and Physics, Leibniz University of Hannover, Appelstrae 11, 30157 Hannover, Germany; 4 Micro and Nanotechnology Graduate Program, TOBB University of Economics and Technology, Sogutozu Caddesi No 43 Sogutozu, 06560 Ankara, Turkey; 5 Department of Physics, Faculty of Science, University of Kurdistan, Sanandaj, 66177-15175 Iran; 6 Department of Physics, Lahore University of Management Sciences, Lahore, Pakistan; 7 Department of Physics, College of Education for Pure Sciences, University of Babylon, Hilla, Iraq; 8 Department of Materials Science and Engineering, Le Quy Don Technical University, Ha Noi, 100000 Vietnam; 9 Department of Electrical and Computer Engineering, Sungkyunkwan University, 16419 Suwon, Korea

**Keywords:** Surfaces, interfaces and thin films, Electronic properties and materials, Electronic structure

## Abstract

$\hbox {Sb}_2\hbox {S}_3$ and $\hbox {Sb}_2\hbox {Se}_3$ are well-known layered bulk structures with weak van der Waals interactions. In this work we explore the atomic lattice, dynamical stability, electronic and optical properties of $\hbox {Sb}_2\hbox {S}_3$, $\hbox {Sb}_2\hbox {Se}_3$ and $\hbox {Sb}_2\hbox {Te}_3$ monolayers using the density functional theory simulations. Molecular dynamics and phonon dispersion results show the desirable thermal and dynamical stability of studied nanosheets. On the basis of HSE06 and PBE/GGA functionals, we show that all the considered novel monolayers are semiconductors. Using the HSE06 functional the electronic bandgap of $\hbox {Sb}_2\hbox {S}_3$, $\hbox {Sb}_2\hbox {Se}_3$ and $\hbox {Sb}_2\hbox {Te}_3$ monolayers are predicted to be 2.15, 1.35 and 1.37 eV, respectively. Optical simulations show that the first absorption coefficient peak for $\hbox {Sb}_2\hbox {S}_3$, $\hbox {Sb}_2\hbox {Se}_3$ and $\hbox {Sb}_2\hbox {Te}_3$ monolayers along in-plane polarization is suitable for the absorption of the visible and *IR* range of light. Interestingly, optically anisotropic character along planar directions can be desirable for polarization-sensitive photodetectors. Furthermore, we systematically investigate the electrical transport properties with combined first-principles and Boltzmann transport theory calculations. At optimal doping concentration, we found the considerable larger power factor values of 2.69, 4.91, and 5.45 for hole-doped $\hbox {Sb}_{{2}}\hbox {S}_{{3}}$, $\hbox {Sb}_{{2}}\hbox {Se}_{{3}}$, and $\hbox {Sb}_{{2}}\hbox {Te}_{{3}}$, respectively. This study highlights the bright prospect for the application of $\hbox {Sb}_2\hbox {S}_3$, $\hbox {Sb}_2\hbox {Se}_3$ and $\hbox {Sb}_2\hbox {Te}_3$ nanosheets in novel electronic, optical and energy conversion systems.

## Introduction

The chalcogenide compounds have attracted great interest owing to their high thermoelectric performance, microelectronics, electronic and optical properties^1–5^. For implementations in all industrial sectors, chalcogenides are presently quite interesting^6^. In this regard, the main technique in the manufacture of two-dimensional (2D) materials is the peeling of layered bulk crystals to produce few-layer flakes or monolayer (single-layer), and it has become the best method in the fabrication of high-quality sheet for several applications^7,8^. There is a large number of monolayers that used in nanodevices, catalysis, field-effect transistors, batteries, hydrogen evolution, and supercapacitors are based on the exfoliated layered materials, for example but not limited to, $\hbox {Bi}_2\hbox {Se}_3$ and $\hbox {Bi}_2\hbox {Te}_3$^9^, $\hbox {MoS}_2$^10^, $\hbox {WS}_2$ and $\hbox {MoSe}_2$^11^, $\hbox {MoTe}_2$^12^, $\hbox {WSe}_2$^13^, CaGe^14^, $\hbox {MnPS}_3$ and $\hbox {MnPSe}_3$^15,16^. The importance of these thin-layer or single-layers is that they can be considered as the starting materials for further manipulation of size and shape to produce custom geometries for nanostructures potentially useful for quantum computers, spintronics, electrothermal computing, and optoelectronics^17–20^.

Meanwhile, the layered semiconductor chalcogenides belonging to the V-VI family has drawn significant attention due to its exceptional properties, such as earth-abundant constituents, low toxicity^21,22^, optical, electronic and thermoelectric properties^23^. According to their semiconducting nature, these material allow overcoming the deficiencies of zero-bandgap in the graphene, showing gorgeous potential for building memory switching^24^, microelectronics, and photovoltaic devices^25,26^. Among semiconductor chalcogenides, antimony-based materials, specifically, the antimony (Sb)- sulphide (S), selenide (Se), telluride (Te) like $\hbox {Sb}_2\hbox {S}_3$, $\hbox {Sb}_2\hbox {Se}_3$, and $\hbox {Sb}_2\hbox {Te}_3$ have drawn extensive attention, which can be considered as binary metal chalcogenide semiconducting materials with high stability. Moreover, the Earth’s crust has an abundance of S, Se, Te and Sb elements of 260, 0.05, 0.005, and 0.2 ppm, respectively^27^. These monolayers possess distinctive crystal structures with semi-one-dimensional ribbons (chains) bound by vdWs, for instance, ($\hbox {Sb}_4\hbox {Se}_6$)n ribbons in $\hbox {Sb}_2\hbox {Se}_3$^28,29^. This type of ribbon-structure contributes to strongly anisotropic charge transport. Besides, $\hbox {Sb}_2\hbox {Te}_3$ has both face-centred cubic and trigonal^30^, and can be found in the liquid state and show high electron density states due to the delocalized electrons at Fermi level^31^. This is disaccorded from the conventional cubic materials with isotropic transport, such as GaAs, Si, Cu(In, Ga)$\hbox {Se}_2$, CdS, and CdSe, and perovskites ($\hbox {CH}_3\hbox {NH}_3\hbox {PbI}_3$)^32–34^. This makes the materials with the like-ribbons structure are preferable in solar cell applications. The $\hbox {Sb}_2\hbox {S}_3$ have drawn considerable attention, and extensively used for photovoltaic applications^35–38^, photodetectors^39^, sodium-ion batteries^40^, and switching^41^ because of its has sufficient elemental storage, tuning of band gap, high current ON/OFF ratio, great dipole moment, higher reversible theoretical capacity, possibility of solution, non-toxicity, mechanical and strong moisture stability at different temperatures^42–46^. $\hbox {Sb}_2\hbox {S}_3$ also shows remarkably an adequate physical criteria for photovoltaic light absorption materials with reasonable efficiencies in power conversion up to 7.5$\%$^47–49^. However, Cai and Chen showed that the comparatively low power conversion of $\hbox {Sb}_2\hbox {S}_3$-based solar cells is mainly as a result of high resistivity of $\hbox {Sb}_2\hbox {S}_3$^50^.

Similarly to $\hbox {Sb}_2\hbox {S}_3$, $\hbox {Sb}_2\hbox {Se}_3$ has recently received great attention to be utilized in applications of batteries^51,52^, photoelectrical^53,54^, thermoelectric devices^55^ and photovoltaic light absorber^56^, due to its prodigious properties such as an optimal bandgap (1-1.3 eV)^57,58^, hole mobility up to 42 $\hbox {cm}^2\hbox {V}^{-1}\hbox {s}^{-1}$^59^, desirable environmental characteristic^60,61^, physiochemical stability^41^, low-cost^62^, and elevated thermoelectric power^24^, as well as interesting optoelectronic features with absorption coefficient larger than 105 $\hbox {cm}^{-1}$ (at short wavelength)^27,63^. Also, a good efficiency in the power conversion up to 9.2$\%$ as very recently reported by Li et al.^64^ On the other hand, $\hbox {Sb}_2\hbox {Te}_3$ is receiving growing research attention within the scientific community because of its gorgeous properties such as low crystallization temperature^65^, and topological insulators^66^. Indeed, $\hbox {Sb}_2\hbox {Te}_3$ chips have already been reported for many applications such as the template materials^65^, lithium-ion batteries^67^, fast memory switching^68^, and thermoelectric devices^69,70^. However, the states of the surface present in the $\hbox {Sb}_2\hbox {Te}_3$ isostructural compounds as the Dirac cone at around the Brillouin zone center ($\Gamma$-point) with a spin texture in charge of fascinating properties like comparative insensitivity to surface information^71,72^. On the other hand, Jiang et al. show that $\hbox {Sb}_2\hbox {Te}_3$ exhibited great characteristics of surface states relevant with Landau level transitions due to their extremely low carrier densities. Besides, the surface states are significantly changed by the asymmetry of the electron-hole from the bulk bands, resulting in the change of the Dirac point and the asymmetry of the band between the and the valence and conduction surface states^73^. Surprisingly, the $\hbox {Sb}_2\hbox {X}_3$ (X= S, Se, Te) were fabricated and experimentally prepared by vacuum thermal evaporation^74–76^, electrodeposition^59^, pulsed laser deposited^77^, spray pyrolysis^78,79^, epitaxy^80^, and chemical deposition^81–83^. These experimental studies have demonstrated that $\hbox {Sb}_2\hbox {X}_3$(X= S, Se, Te) can be efficiently used as potential material for various applications.

Despite the hexagonal $\hbox {Sb}_2\hbox {X}_3$ (X = S, Se, Te) monolayers were under comprehensive researches over the past years based on the encouraging reports on their excellent properties, the physical properties of novel orthorhombic $\hbox {Sb}_2\hbox {X}_3$ (X = S, Se, Te) monolayers still undiscovered. Hence, we investigated in the present work the structural, electronic, optical, thermoelectric properties ofnovel $\hbox {Sb}_2\hbox {X}_3$ (X = S, Se, Te) monolayers crystallize in the orthorhombic structures by the density functional theory (DFT). Furthermore, for many related uses, the properties reported in this research may enable engineers and technicians to design and manufacture special types of modern nanoelectronics and optoelectronics devices.

## Method

The density-functional theory (DFT) calculations in this work are performed using the plane-wave basis projector augmented wave (PAW) method along with generalized gradient approximation (GGA) with Perdew-Burke-Ernzerhof(PBE)^84,85^ functional as implemented in the Vienna *ab-initio* Simulation Package (VASP)^86,87^. Moreover, for the band structure calculations spin-orbit-coupling (SOC) was included on top of GGA and Heyd-Scuseria-Ernzerhof (HSE06)^88^ screened-nonlocal-exchange functional of the generalized Kohn-Sham scheme, respectively for more accurate band gap calculations. The kinetic energy cut-off of 500 eV was set for plane-wave expansion and the energy was minimized structures are obtained until variation in the energies fall below 10$^{-8}$ eV. Van der Waals (vdW) correction proposed by Grimme to describe the long-range vdW interactions^89^. Charge transfers analysis is accomplished using the Bader technique^90^. To get optimized structures, total Hellmann-Feynman forces were reduced to 10$^{-7}$ eV/Å. $21 \times 21\times$1 $\Gamma$ centered *k*-point sampling was used or the primitive unit cells by using Monkhorst-Pack^91^. In this work, the phonon dispersion relations are acquired using machine-learning interatomic potentials on the basis of moment tensor potentials (MTPs)^92^. The training sets are prepared by conducting ab-initio molecular dynamics (AIMD) simulations over $4 \times 2\times$1 supercells with $2 \times 2\times$1 k-point grids and a time step of 1 fs. AIMD simulations are carried out at 50 and 600 K, each for 800 time steps and half of the full trajectories are selected to create the training sets. MTPs were then passively fitted using the methodology explained in the previous works^93,94^. The PHONOPY code^95^ is employed to obtain phonon dispersion relations and harmonic force constants over $4 \times 12\times$1 supercells using the trained MTPs for the interatomic force calculations^93,94^. The optical properties, such as imaginary and real parts of dielectric tensor (Im($\varepsilon$) and Re($\varepsilon$)), absorption coefficient ($\alpha$), reflectivity (R) Random phase approximation (RPA)$^{?}$ method on the basis of screened hybrid Heyd-Scuseria-Ernzerhof functional (HSE06)^88^ was employed to study optical properties using the VASP^86,87^. The optical properties were evaluated using a dense k-point grid of $18 \times 8\times$1 $\Gamma$-centered Monkhorst-Pack^91^. For more details about calculations of optical properties see supporting information. The electrical transport coefficients, such as electrical conductivity ($\sigma$), Seebeck coefficient (*S*), and electronic thermal conductivity ($\kappa _{e}$) are calculated using the Boltzmann transport equation as implemented in the Boltztrap2 code^96^ under the constant relaxation time and rigid band approximations.

## Structural properties

**Figure 1:**
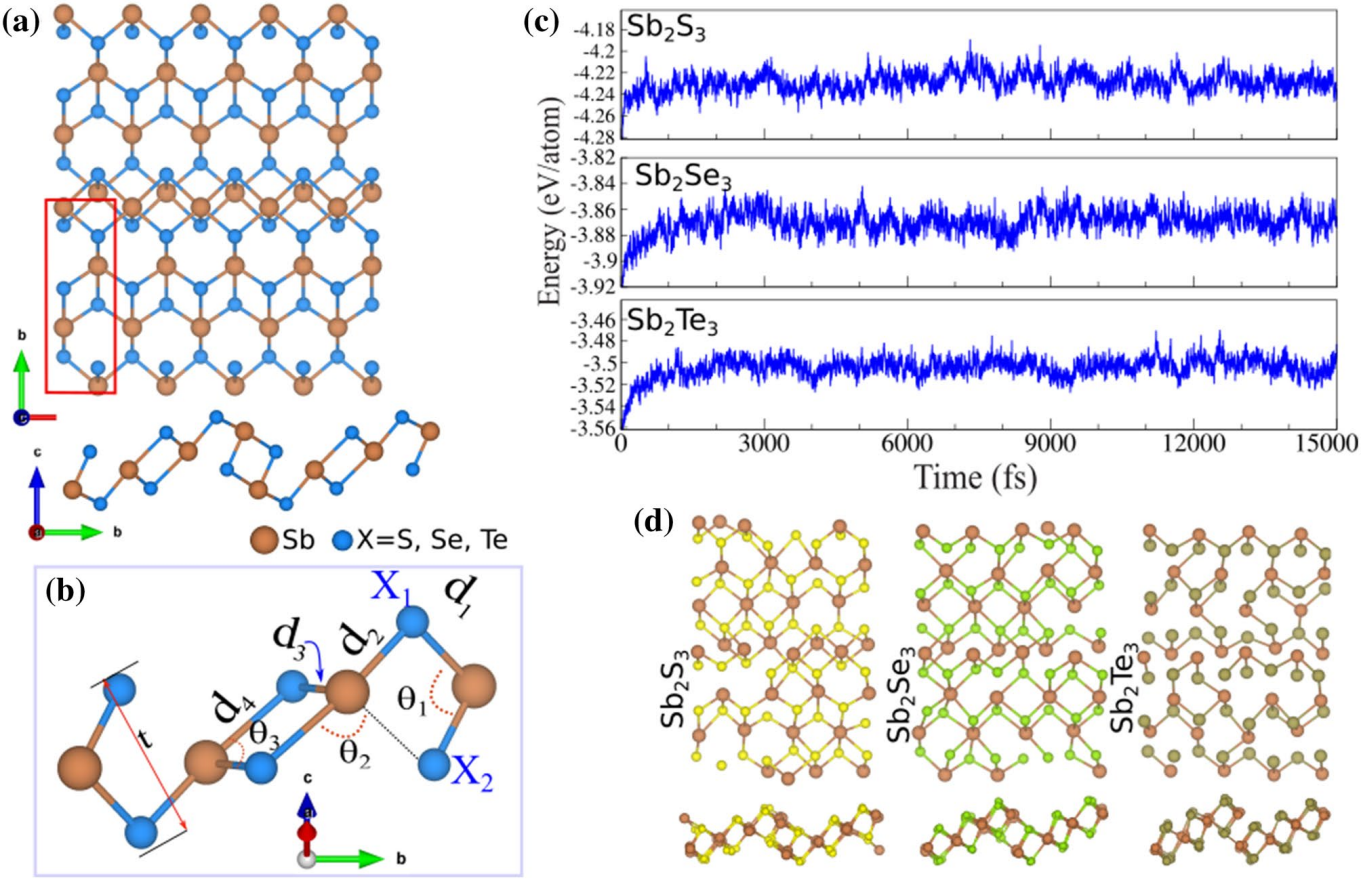
(**a**) Different views of atomic structure of $\hbox {Sb}_2\hbox {X}_3$ (X = S, Se, Te) monolayer, with the unit cell distinguished with a rectangle. (**b**) Schematic of structural parameters in a $\hbox {Sb}_2\hbox {X}_3$ lattice. (**c**) Ab initio molecular dynamics (AIMD) for these monolayers at room temperature. (**d**) The top and side views of the structures after 5 ps of simulation.

The geometrical atomic structures of $\hbox {Sb}_2\hbox {X}_3$ (X = S, Se, Te) monolayers in the different views are depicted in Fig. 1a. The primitive unit cell of the $\hbox {Sb}_2\hbox {X}_3$ monolayers is indicated by red rectangular and is formed by 10 atoms with space group *Pmcn*. In the crystal structure of $\hbox {Sb}_2\hbox {X}_3$, each Sb atom is encompassed by six X (X = S, Se, Te) atoms and each X atom is encompassed by four Sb atoms. Notice that the vectors $\overrightarrow{a}$ $\ne$ $\overrightarrow{b}$ are the translational unit cell vectors. The calculated lattice parameters of $\textit{a}$ ($\textit{b}$) in the $\hbox {Sb}_2\hbox {S}_3$, $\hbox {Sb}_2\hbox {Se}_3$ and $\hbox {Sb}_2\hbox {Te}_3$ monolayers are equal to 3.86 (10.92), 3.92 (9.99) and 3.87 (9.65) Å, respectively, as listed in Table 1. Notice that the bond lengths $d_{1,2}$ and $d_{3,4}$ are determined to be 2.66/2.59 Å and 2.56/4.94 Å for $\hbox {Sb}_2\hbox {S}_3$ monolayer, 2.75/2.90 Å and 2.77/4.97 Å for $\hbox {Sb}_2\hbox {Se}_3$ monolayer, 2.95/3.13 Å and 2.99/3.02 Å for $\hbox {Sb}_2\hbox {Te}_3$ monolayer, respectively. The two angles of X-Sb-X in lattice of $\hbox {Sb}_2\hbox {S}_3$, $\hbox {Sb}_2\hbox {Se}_3$ and $\hbox {Sb}_2\hbox {Te}_3$ are 91/106/86$^{\circ }$, 95/90/92$^{\circ }$ and 97/86/94$^{\circ }$, respectively, which result in high anisotropic lattice. The thickness of $\hbox {Sb}_2\hbox {S}_3$, $\hbox {Sb}_2\hbox {Se}_3$ and $\hbox {Sb}_2\hbox {Te}_3$ monolayers are calculated to be 3.17, 3.66 and 3.79 Å, respectively.

**Table 1:** Structural and electronic parameters of $\hbox {Sb}_2\hbox {X}_3$ (X = S, Se, Te) monolayers as shown in Fig. 1b, including lattice constants $\mathbf{a},b$; the bond lengths between Sb-X atoms $d_{1,2,3,4}$; the bond angles between X-Sb-X atoms $\theta _{1,2,3}$; the thickness defined by the difference between the largest and smallest z coordinates of X atoms (*t*); the cohesive energy per atom, $(E_{coh})$; the charge transfer $(\Delta {Q})$ between atoms Sb and $X_{1}$ ($X_{2}$) atoms are shown inside (outside) parentheses as shown in Fig. 1b; the work function $(\Phi ).$

	*a (b)* (Å)	${d}_{1/2}$ (Å)	d$_{3/4}$ (Å)	*t* (Å)	$\theta _{1/2/3}$ ($^{\circ }$)	$E_{coh}$ (eV/atom)	$\Delta {Q}$ (e)	$\Phi$ (eV)	$E_{g}$ (eV)
$\hbox {Sb}_2\hbox {S}_3$	3.86 (10.92)	2.66/2.59	2.56/4.94	3.17	91/106/86	− 7.94	0.75 (0.82)	5.17	1.22 (2.15)
$\hbox {Sb}_2\hbox {Se}_3$	3.92 (9.99)	2.75/2.90	2.77/4.97	3.66	95/90/92	− 7.36	0.59 (0.64)	4.94	0.96 (1.35)
$\hbox {Sb}_2\hbox {Te}_3$	3.87 (9.65)	2.95/3.13	2.99/3.02	3.79	97/86/94	− 6.81	0.36 (0.37)	4.53	0.86 (1.37)

The band gap $(E_{g})$ of PBE and HSE06 are shown outside and inside parentheses, respectively.

The difference charge density ($\Delta \rho$) is defined as:

1 $$\begin{aligned} \Delta \rho =\rho _{tot}-\rho _{X}-\rho _{Sb} \end{aligned}$$

where $\rho _{tot}$, $\rho _{X}$ and $\rho _{Sb}$ show charge densities of the $\hbox {Sb}_2\hbox {X}_3$ and isolated atoms, respectively. It is clear that Sb atoms are positively charged and surrounded by negatively charged S, Se or Te atoms. Each S, Se and Te atom labeled *X*1(*X*2) (see Fig. 1b), gains about 0.75*e* (0.82*e*), 0.59*e* (0.64*e*) and 0.36*e* (0.37*e*) from the adjacent Sb atoms in $\hbox {Sb}_2\hbox {S}_3$, $\hbox {Sb}_2\hbox {Se}_3$ and $\hbox {Sb}_2\hbox {Te}_3$, respectively. Worthy to note that the charge redistribution stem from different electro-negativities of 2.05 (Sb), 2.58 (S), 2.55 (Se) and 2.1 (Te).

Cohesive energy, which is defined as the energy required to separate condensed material into isolated free atoms, is one of the most important physical parameters in quantifying the energetic stability of materials. The cohesive energy per atom is calculated using the following equation:

2 $$\begin{aligned} E_{coh} = \frac{E_{tot}-3E_{X}-2E_{Sb}}{n_{tot}} \end{aligned}$$

where $E_{X}$ and $E_{Sb}$ represent the energies of isolated single X (S, Se and Te) and Sb atoms, $\hbox {n}_{{tot}}$ is the total number of atoms in unit cell. $E_{tot}$ represents the total energy of the $\hbox {Sb}_2\hbox {X}_3$ monolayer. The cohesive energy of $\hbox {Sb}_2\hbox {S}_3$ and $\hbox {Sb}_2\hbox {Se}_3$ are found to be -7.94 and -7.36 eV/atom, respectively. While the cohesive energy of $\hbox {Sb}_2\hbox {Te}_3$ is -6.81 eV/atom. These finding indicates that the formation of $\hbox {Sb}_2\hbox {S}_3$ is more favorable than the others. The results of Ab initio molecular dynamics (AIMD) simulation for the studied monolayers at room temperature are shown in Fig. 1c. The snapshots of top and side views of the structures after 5 ps are illustrated in Fig. 1d. Analysis of the AIMD trajectories also shows that the structure could stay intact at 500 K with very stable energy and temperature profiles, proving the thermal stability of the $\hbox {Sb}_2\hbox {X}_3$ monolayer.

**Figure 2:**
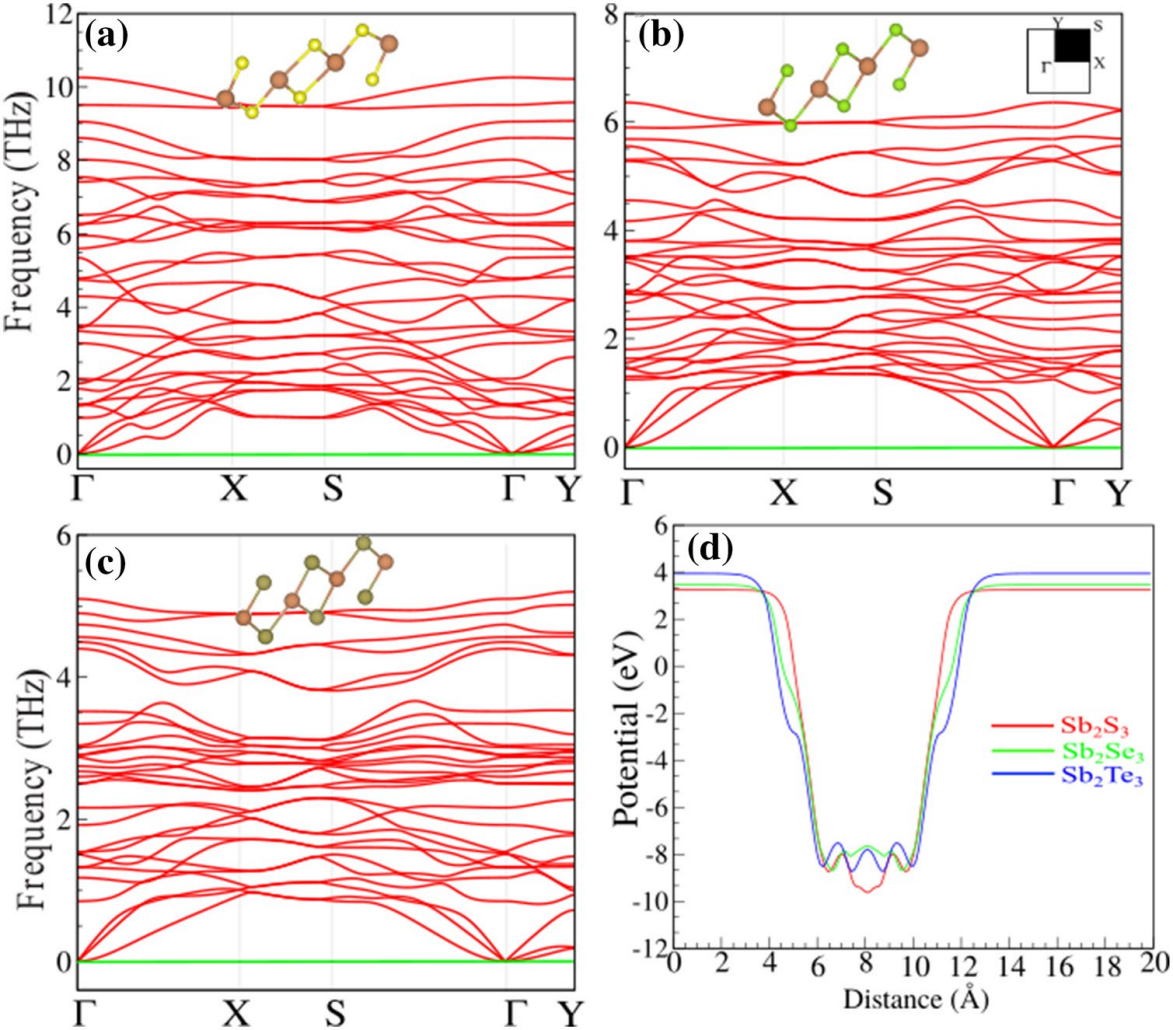
Phonon dispersions of (**a**) $\hbox {Sb}_2\hbox {S}_3$, (**b**) $\hbox {Sb}_2\hbox {Se}_3$ and (**c**) $\hbox {Sb}_2\hbox {Te}_3$ monolayers. Optimized atomic structure indicated as inset. (**d**) Electrostatic potential for the $\hbox {Sb}_2\hbox {X}_3$ monolayers.

Apparently, phonon branches are free from any imaginary frequencies indicating the dynamical stability of the structures. The more negative values for cohesive energies suggest that the energetically more stable monolayer, and the structures represent more stability when the atoms get lighter. The dynamical stability of single-layers of $\hbox {Sb}_2\hbox {X}_3$ is verified by calculating their phonon band dispersions through the whole BZ which are presented in Fig. 2a–c.

The electrostatic potential for the $\hbox {Sb}_2\hbox {X}_3$ monolayers is shown in Fig. 2d. Notice that the electrostatic potential of studied monolayers are flat in the vacuum region. The work function was calculated using the following $\Phi =E_{vacuum}-E_{F}$, where $E_{vacuum}$ is the energy of the vacuum which is extracted from the electrostatic potential, and $E_{F}$ is the Fermi energy. The calculated work function of the studied monolayers are 5.17 ($\hbox {Sb}_2\hbox {S}_3$), 4.94 ($\hbox {Sb}_2\hbox {Se}_3$) and 4.53 eV ($\hbox {Sb}_2\hbox {Te}_3$). We found that the work function is decreases as the electronegativity of X (X = S, Se and Te) atom decreases.

## Electronic properties

**Figure 3:**
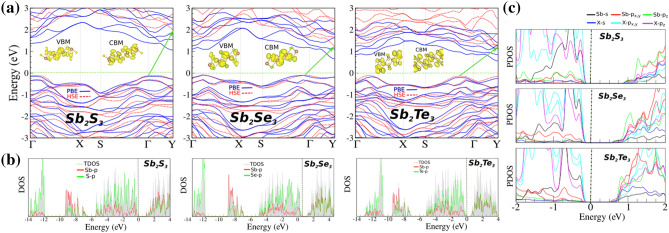
(**a**) Electronic band structure, (**b**) density of states (DOS) and (**c**) projected DOS (PDOS) of $\hbox {Sb}_2\hbox {X}_3$ monolayers. The zero of energy is set to Fermi-level.

The electronic band structure of $\hbox {Sb}_2\hbox {X}_3$ monolayers are shown in Fig. 3a. Our results show that, $\hbox {Sb}_2\hbox {S}_3$ is an indirect semiconductor with a band gap of 1.22 eV within PBE functional. Notice that the valance band minimum (VBM) is located at the $\Gamma$ point, while the conduction band maximum (CBM) is located along the $\Gamma$-S points. Similar $\hbox {Sb}_2\hbox {S}_3$, $\hbox {Sb}_2\hbox {Se}_3$ and $\hbox {Sb}_2\hbox {Te}_3$ exhibit semiconducting characteristics with indirect band gap of 0.96 eV and 0.86 eV, respectively. Notice that, we can see that both the VBM and CBM of these monolayers are located along the $\Gamma$ and Y points, respectively. The electronic band structure of $\hbox {Sb}_2\hbox {X}_3$ monolayers with considering spin orbital coupling (SOC) are shown in Fig. S1a–c in the supplementary information (SI). With considering of SOC effect, the band gaps of the $\hbox {Sb}_2\hbox {S}_3$, $\hbox {Sb}_2\hbox {Se}_3$ and $\hbox {Sb}_2\hbox {Te}_3$ monolayers decrease to 0.95, 0.75 and 0.45 eV, respectively. The charge densities of the VBM and CBM orbitals are shown in the inset (see inset in Fig. 3a). It is clear that energy bands around the Fermi-level are formed mainly by X atoms. Since these monolayers are semiconductor, the HSE06 functional was also used to study the electronic band structures, shown in Fig. 3. It is clear that the HSE06 results are consistent with PBE/GGA for the type of indirect semiconducting band gap in these systems. Based on the acquired band structure by HSE06 method, the indirect band gap of $\hbox {Sb}_2\hbox {S}_3$, $\hbox {Sb}_2\hbox {Se}_3$ and $\hbox {Sb}_2\hbox {Te}_3$ was estimated to be 2.15, 1.35 and 1.37 eV, respectively. The band gap value of $\hbox {Sb}_2\hbox {Te}_3$ is still larger than that reported in Ref.^97^. The nature of such difference is due to the underestimation of traditional DFT method. Therefore, our calculations methods are reliable. In order to explain the origin of the electronic states, the DOS and the PDOS are shown in Fig. 3b,c, respectively. It is clearly seen that the semiconducting character of $\hbox {Sb}_2\hbox {S}_3$ comes from S and Te atoms, while Sb atoms does now show any contribution. From DOS and PDOS, it is clearly seen that the VBM are composed of the $p_z$ and $p_{x,y}$ orbitals states of S atom, while the CBM comes from $p_{z}$ and $p_{x,y}$ orbitals of S and Sb atoms. We found that the VBM of $\hbox {Sb}_2\hbox {Se}_3$ and $\hbox {Sb}_2\hbox {Te}_3$ originates from Se/Te-$p_{x,y}$ orbitals, while the CBM consists of Se/Te-$p_{z}$ and Sb-$p_{z}$ orbital states.

## Optical properties

**Figure 4:**
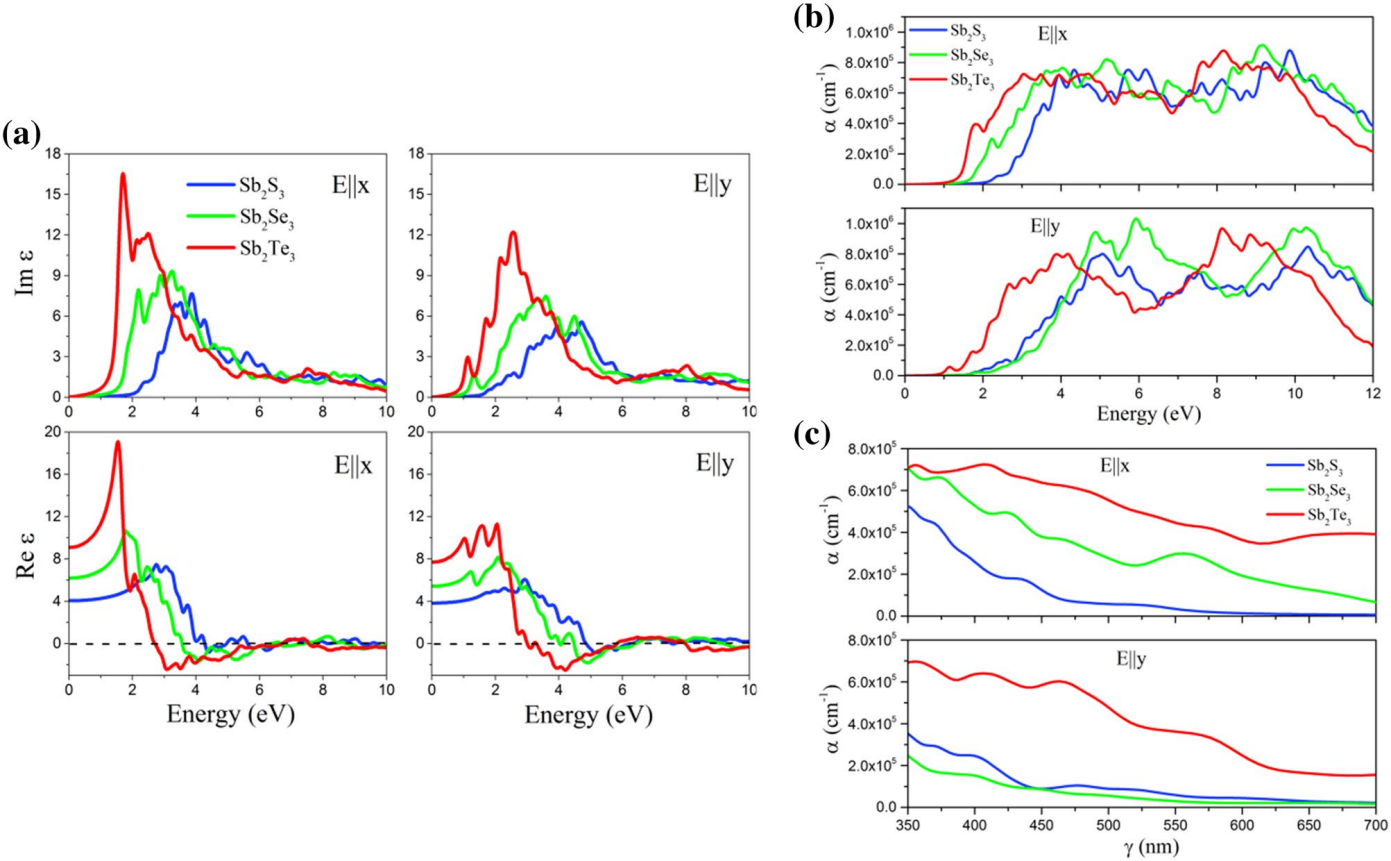
(**a**) Imaginary and real parts of the dielectric function as a function of photon energy of the $\hbox {Sb}_2\hbox {S}_3$, $\hbox {Sb}_2\hbox {Se}_3$ and $\hbox {Sb}_2\hbox {Te}_3$ monolayers for the in-plane polarizations ($\hbox {E}\parallel \hbox {x}$ and $\hbox {E}\parallel \hbox {y}$), predicted using the RPA + HSE06 approach. Absorption coefficient as a function of (**b**) wavelength and (**c**) energy for the $\hbox {Sb}_2\hbox {X}_3$ monolayers for the in-plane polarizations ($\hbox {E}\parallel \hbox {x}$ and $\hbox {E}\parallel \hbox {y}$) in the UV–vis range of light, predicted using the RPA + HSE06 approach.

Now we discuss the optical responses of this novel 2D system using the RPA+ HSE06. The depolarization effect of 2D materials along out-of-plane direnction is strong^98^, hence we only report the optical properties for in-plane polarizations ($\hbox {E}\parallel \hbox {x}$ and $\hbox {E}\parallel \hbox {y}$). Due to the asymmetric lattice along the x- and y-directions the optical properties are aisotropic for light polarizations along these axes and hence the optical properties along both directions are reported. Fig. 4a illustrates the imaginary and real parts of the dielectric function of these 2D systems along the in-plane directions. It can be seen that the Im($\varepsilon$) along x- and y-axes starts with a gap confirming the semiconducting properties for optical spectra along these directions for these novel 2D systems. The first peak of Im($\varepsilon$) occurs at 2.39, 2.16 and 1.67 eV for the $\hbox {Sb}_2\hbox {S}_3$, $\hbox {Sb}_2\hbox {Se}_3$ and $\hbox {Sb}_2\hbox {Te}_3$ monolayers, respectively, along x-axis while it appears at 1.74, 1.36 and 1.10 eV along y-axis. These results indicate that the first peaks of Im($\varepsilon$) for all monolayer systems are in visible and *IR* range of light along talong x- and y-axes. These results also indicate that by increasing atomic number of X element in $\hbox {Sb}_2\hbox {X}_3$ monolayers, the first Im($\varepsilon$) peak slightly shifts to lower energies (red shift). The static dielectric constants (the values of Re($\varepsilon$) at zero energy) for $\hbox {Sb}_2\hbox {Te}_3$ monolayer along $\hbox {E}\parallel \hbox {x}$ were calculated to be 4.0, 6.4 and 9.1, respectively, while the corresponding values for $\hbox {E}\parallel \hbox {y}$ are 3.9, 5.5 and 7.8. The plasma frequencies which define by the roots of Re($\varepsilon$) with x = 0 line^99,100^ were calculated for these 2D monolayers. The values of first plasma frequencies along x-axis are 4.27, 3.51 and 2.65 eV for $\hbox {Sb}_2\hbox {S}_3$, $\hbox {Sb}_2\hbox {Se}_3$ and $\hbox {Sb}_2\hbox {Te}_3$ monolayers, respectively, while the corresponding values for the same systems along $\hbox {E}\parallel \hbox {y}$ are 4.8, 4.45 and 2.98 eV. The absorption coefficient $\alpha$ for all studied 2D systems along in-plane polarization are shown in Fig. 4b,c. The first absorption peaks for the $\hbox {Sb}_2\hbox {S}_3$, $\hbox {Sb}_2\hbox {Se}_3$ and $\hbox {Sb}_2\hbox {Te}_3$ monolayers along $\hbox {E}\parallel \hbox {x}$ are in the visible range of light and occur at energy of 2.39, 2.18 and 1.77 eV, respectively. The corresponding values of the first absorption peaks along y-axis locate at energy of 1.98, 2.13 and 1.14 eV for the same monolayers. These results show the first absorption peaks of $\hbox {Sb}_2\hbox {S}_3$ and $\hbox {Sb}_2\hbox {Se}_3$ monolayers for $\hbox {E}\parallel \hbox {y}$ are in visible range of light while it occur at *IR* range for $\hbox {Sb}_2\hbox {Te}_3$ monolayer. According to our optical results, these 2D systems have potential applications in optoelectronic devices in the visible and *IR* spectral range. Fig. 4b illustrates the absorption coefficient as a function of wavelength for the $\hbox {Sb}_2\hbox {X}_3$ monolayers for the in-plane polarizations in the UV-vis range (350-700 nm) of light. It is obvious that the absorption coefficients for these 2D materials are high ($\sim$10 $^5\hbox {cm}^{-1}$) to be used in optical devices^101^. Interestingly, optically anisotropic character of these systems along x- and y-axes is highly desirable for the design of polarization-sensitive photodetectors^102^.

## Thermoelectric properties

**Figure 5:**
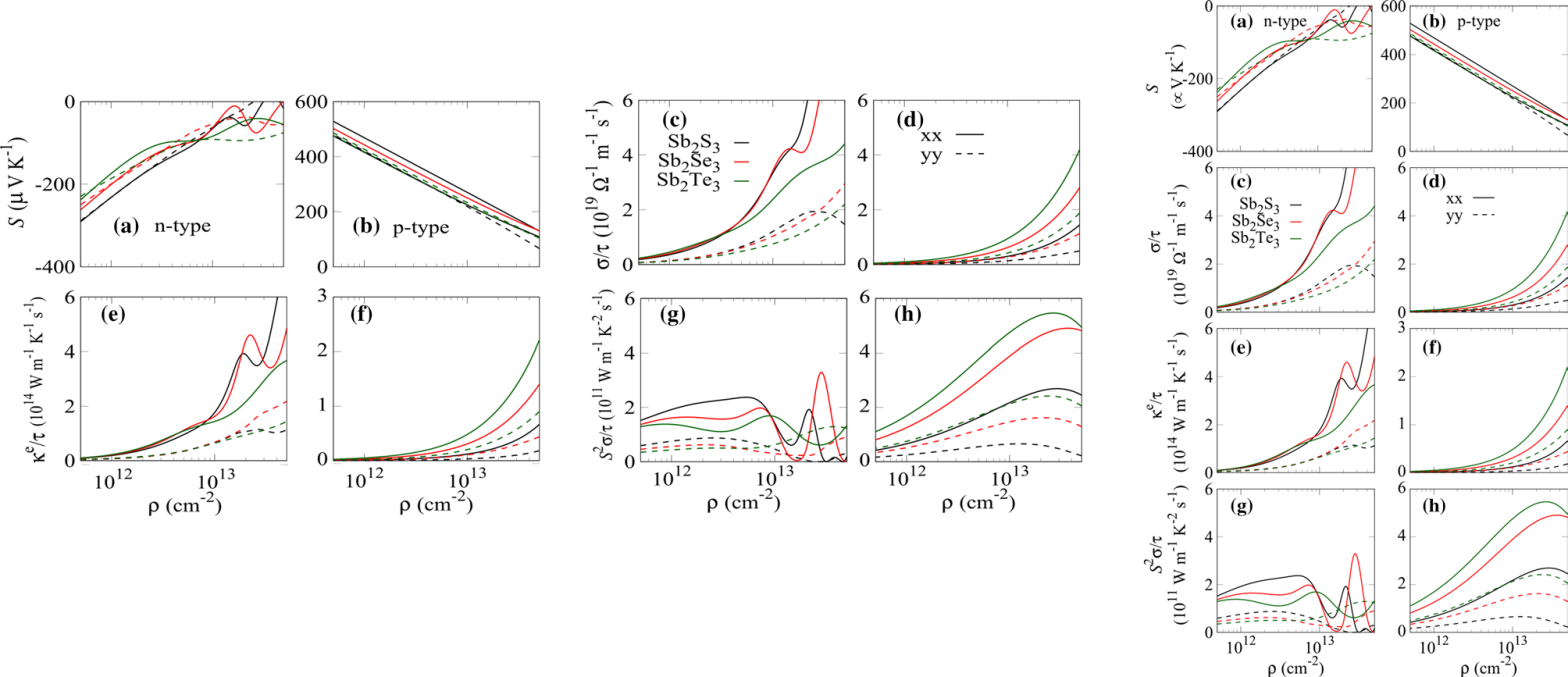
The electrical transport properties as function carrier concentration at 300 K of the $\hbox {Sb}_2\hbox {X}_3$ monolayers (**a**,**b**) Seebeck coefficient, (**c**,**d**) electrical conductivity, (**e**,**f**) electronic thermal conductivity, and (**g**,**h**) power factor. The solid and dashed lines represent the x-and y-direction, respectively.

The Seebeck coefficients as a function of carrier concentration for $\hbox {Sb}_{{2}}\hbox {X}_{{3}}$ monolayers are presented in Fig. 5a,b. Large Seebeck coefficients are found for the *p*-type doping in these monolayers due to the flat valence band which increases the density of states near the Fermi level. Monolayer $\hbox {Sb}_{{2}}\hbox {S}_{{3}}$ achieves higher Seebeck coefficient values of 530 $\mu \hbox {VK}^{-1}$, 483 $\mu \hbox {VK}^{-1}$ at 300 K along the *x* and *y* directions, respectively. The variation in electrical conductivity ($\sigma /\tau$) and the electronic thermal conductivity ($\kappa ^{e}/\tau$ ) with respect to carrier concentration are plotted in Fig. 5c–f. The $\sigma /\tau$ and $\kappa ^{e}/\tau$ of *n*-type are larger than that of the *p*-type one at the same doping level because of the dispersive conduction bands which lower the effective mass. The $\sigma /\tau$ and $\kappa ^{e}/\tau$ follow the Wiedemann–Franz law. The $\sigma /\tau$ exhibits anisotropic behavior where the $\sigma /\tau$ value along the x-direction is higher than that alone the y-direction because of the dispersive band nature along $\Gamma$-X than $\Gamma$-Y direction. The power-factor (PF) ($S^{2}\sigma /\tau$) is obtained using the calculated Seebeck coefficient and electrical conductivity as shown in Fig. 5g,h. For *p*-type monolayer $\hbox {Sb}_{{2}}\hbox {Te}_{{3}}$, the maximum *PF* values of 5.45 and 2.44 ($10^{11}$ $\hbox {Wm}^{-1}\hbox {K}^{-2}\hbox {s}^{-1}$) are obtained at 300 K along the *x* and *y* directions. The value of *PF* is higher for the p-type doping because of large Secbeck coefficients. These values are significantly larger, demonstrating great potential as a promising candidate for thermoelectricity.

## Conclusion

In summary, we introduced $\hbox {Sb}_2\hbox {X}_3$ (X = S, Se, and Te) monolayers as novel, dynamically and thermally stable 2D indirect gap semiconductors. Using the HSE06 method the band gaps of $\hbox {Sb}_2\hbox {S}_3$, $\hbox {Sb}_2\hbox {Se}_3$ and $\hbox {Sb}_2\hbox {Te}_3$ monolayers are predicted to be 2.15, 1.35 and 1.37 eV, respectively, appealing for applications in nanoelectronics. Optical calculations indicate that the first absorption peaks of these novel nanosheets along in-plane polarization are located in IR and visible range of light, suggesting its prospect for applications in optoelectronics. Moreover, the in-plane optical anisotropy of these novel 2D materials is highly desirable for the design of polarization-sensitive photodetectors. We also show that $\hbox {Sb}_{{2}}\hbox {X}_{{3}}$ monoalyers can be used for thermoelectric application because of their larger power factors, the power factor for the hole-doped $\hbox {Sb}_{{2}}\hbox {Te}_{{3}}$ can reach 5.45 ($10^{11}\hbox {Wm}^{-1}\hbox {K}^{-2}\hbox {s}^{-1}$). Our results confirm the stability and highlights the outstanding prospect for the application of $\hbox {Sb}_2\hbox {X}_3$ nanosheets in novel electronic, optical and energy conversion systems.

## Supplementary Information

Supplementary Information.
