# Supplementary material for: Ab initio prediction of semiconductivity in a novel two-dimensional Sb2X3 (X= S, Se, Te) monolayers with orthorhombic structure
Source: Sci Rep. 2021 May 14;11:10366. doi: 10.1038/s41598-021-89944-4 (PMC8121886; doi:10.1038/s41598-021-89944-4)
Supplement: Supplementary file 1 — Supplementary Information. [file 41598_2021_89944_MOESM1_ESM.doc]

**Supporting information**

**Ab initio prediction of semiconductivity in a novel two-dimensional Sb_2_X_3_ (X= S, Se, Te) monolayers with orthorhombic structure**

**A. Bafekry^1,2,*^, B. Mortazavi^3^, M. Faraji^4^, M. Shahrokhi^5^, A. Shafique^6^, H. R. Jappor^7^, Chuong V. Nguyen^8^, M. Ghergherehchi^9,*^, S.A.H. Feghhi^10^**

^1^Department of Radiation Application, Shahid Beheshti University, Tehran, Iran
^2^Department of Physics, University of Antwerp, Groenenborgerlaan 171, B-2020 Antwerp, Belgium
^3^Chair of Computational Science and Simulation Technology, Institute of Photonics, Department of Mathematics and Physics, Leibniz University of Hannover, Appelstrae 11,30157 Hannover, Germany
^4^Micro and Nanotechnology Graduate Program, TOBB University of Economics and Technology, Sogutozu Caddesi No 43 Sogutozu, 06560, Ankara, Turkey
^5^Department of Physics, Faculty of Science, University of Kurdistan, 66177-15175 Sanandaj, Iran
^6^Department of Physics, Lahore University of Management Sciences,Lahore, Pakistan
^7^Department of Physics, College of Education for Pure Sciences, University of Babylon, Hilla, Iraq
^8^Department of Materials Science and Engineering, Le Quy Don Technical University, Ha Noi 100000, Vietnam
^9^College of Electronic and Electrical Engineering, Sungkyunkwan University, Suwon, Korea
^10^Department of Radiation Application, Shahid Beheshti University, Tehran, Iran
*Corresponding authors: Email: [bafekry.asad@gmail.com](mailto:bafekry.asad@gmail.com) and [mitragh@skku.edu](mailto:mitragh@skku.edu)

**Computational details for the optical calculations**

In the random phase approximation, the imaginary part of the interband dielectric permittivity is given by [1]:

$\text{Im}\varepsilon_{\alpha\beta}(\omega)=\frac{4\pi^{2}e^{2}}{\Omega}Lim_{q\to0}\frac{1}{q^{2}}\sum_{c,\upsilon,k} 2\omega_{k}\delta(\varepsilon_{ck}-\varepsilon_{vk}-\omega)\left\langle u_{ck+e_{\alpha}q}\left|\left.u_{vk}\right\rangle\right.\right.\left\langle u_{ck+e_{\beta}q}\left|u_{vk}\right.\right\rangle$ (1)

where *q* is the Bloch vector of the incident wave, *w_k_*is the **k**-point weight and the band indices *c* and *v* are restricted to the conduction and the valence band states, respectively. By using the$\text{Im}\varepsilon_{\alpha\beta}(\omega)$, one can determine the corresponding real part via the Kramers–Kronig relations:

$\text{Re}\varepsilon_{\alpha\beta}(\omega)=1+\frac{2}{\pi}P\int\frac{\omega^{'}\text{Im}\varepsilon_{\alpha\beta}(\omega^{'})}{(\omega^{'})^{2}-\omega^{2}+i\eta}d\omega^{'}$ (2)

where *P* denotes the principle value and *η* is the complex shift.

The adsorption coefficient determined as:

$a_{\alpha\beta}(\omega)=\frac{2\omega k_{\alpha\beta}(\omega)}{c}$ (3)

where *k_αβ_* is imaginary part of the complex refractive index and *c* is the speed of light in vacuum, known as the extinction index. It is given by the following relations

$k_{\alpha\beta}(\omega)=\sqrt{\frac{\left|\varepsilon_{\alpha\beta}(\omega)-\text{Re}\varepsilon_{\alpha\beta}(\omega)\right|}{2}}$ (4)

The reflectivity is given by

$R_{ij}(\omega)=\frac{(n-1)^{2}+k^{2}}{(n+1)^{2}+k^{2}}$ (5)

where *n* and *k* are real and imaginary parts of the complex refractive index, which are known as the refractive index and the extinction index, respectively.


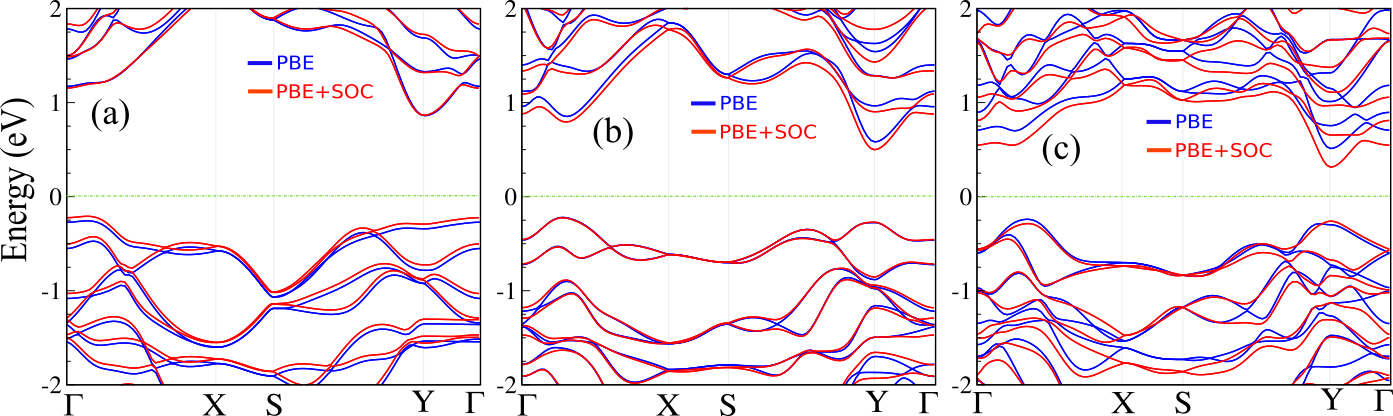
Fig. S1. Electronic band structure of the (a) Sb_2_S_3_ , (b) Sb_2_Se_3_ and (c) Sb_2_Te_3_ monolayers with/without considering SOC.
